# Relative time in physical activity and sedentary behaviour across a 2-year pedometer-based intervention in people with prediabetes or type 2 diabetes: a secondary analysis of a randomised controlled trial

**DOI:** 10.1186/s44167-023-00020-w

**Published:** 2023-06-01

**Authors:** Kristina Larsson, Philip Von Rosen, Jenny Rossen, Unn-Britt Johansson, Maria Hagströmer

**Affiliations:** 1 Department of Health Promoting Science, Sophiahemmet University, P.O. Box 5605, 114 86 Stockholm, Sweden; 2 Division of Physiotherapy, Department of Neurobiology, Care Sciences and Society, Karolinska Institutet, Stockholm, Sweden; 3 Department of Clinical Science and Education, Södersjukhuset, Karolinska Institutet, Stockholm, Sweden; 4 Academic Primary Care Center, Region Stockholm, Stockholm, Sweden

**Keywords:** Intervention, Movement behaviour, Relative time, Steps

## Abstract

**Background:**

People with prediabetes or type 2 diabetes (T2D) need to be physically active, including moderate-to-vigorous intensity physical activity (MVPA) and light-intensity physical activity (LIPA) and reduce time in sedentary behaviour (SB). Few studies have evaluated the effect of randomised controlled trials taking all movement behaviours into account. This study aimed to investigate the effects of a 2-year pedometer-based intervention in people with prediabetes or T2D on relative time in movement behaviours.

**Methods:**

Secondary analysis of longitudinal data on individuals with prediabetes or T2D from a three-armed randomised controlled trial, the Sophia Step Study, was conducted. The three groups were (1) a multi‑component group (self‑monitoring of steps with a pedometer plus counselling), (2) a single‑component group (self‑monitoring of steps with a pedometer, without counselling), and (3) a standard care group (control). The three behaviours MVPA, LIPA and SB during waking hours were measured with an ActiGraph GT1M accelerometer at baseline, 6, 12, 18 and 24 months. Relative time in MVPA, LIPA and SB for each participant at each time point was calculated and used as outcome measures. Linear mixed models assessed the effect of the intervention over time.

**Results:**

In total 184 participants with mean (SD) age 64.3 (7.6) years and 41% female was included. In the multi-component group, compared to the control group, a significant group-by-time interaction effect for relative time in all three behaviours was found at 6 and 18 months and for MVPA and SB at 24 months. In the single-component group, compared to the control group, an effect occurred in the MVPA and SB behaviours at 6 months and MVPA and LIPA at 24 months. The estimated marginal means ranged from 0.9 to 1.5% of more MVPA, 1.9–3.9% of less LIPA and from 0.5% of less SB to 1.7 more SB in the intervention groups compared to the control group.

**Conclusions:**

The findings show a beneficial effect on all behaviours over time in the two intervention groups compared to the control group. A more pronounced effect occurred in the multi-component intervention compared to the single-component intervention, implicating the importance of counselling in pedometer-based interventions.

*Trial registration* ClinicalTrials.gov, NCT02374788

## Background

The prevalence of prediabetes and type 2 diabetes (T2D) is rising globally [1]. Regular physical activity is associated with preventing and controlling the disease [2, 3]. However, most people with prediabetes or T2D do not meet recommended physical activity levels [4–6]. One way to reach this patient group is by using primary care as an arena [7], together with interventions using pedometers as a motivational tool. Pedometers has been shown to have a positive short-term effect on increasing physical activity [8–13]. For people with T2D, the intensity of physical activity seems especially relevant. Time in moderate-to-vigorous intensity physical activity (MVPA) and light-intensity physical activity (LIPA) is linked to better cardiovascular risk profiles, while the opposite occurs in sedentary behaviour (SB) [14]. In addition, a decrease in prolonged SB and an increase in MVPA are associated with reduced HbA1c levels [15].

Accelerometers, which generate data in different intensities, are commonly used to assess physical activity [16]. The MVPA, LIPA and SB intensities can be expressed as interdependent movement behaviours, i.e., if time in one movement behaviour increases, time in other movement behaviours decreases, given that time is an invariant quantity. Analysing movement behaviours as separate isolated behaviours, with each behaviour in absolute time, is the most commonly applied method. However, analysis methods with relative time in each behaviour in relation to the other movement behaviours should be considered [17, 18]. Using absolute time can lead to an incomplete picture of movement behaviours. Also, more studies using relative time in different populations (e.g., T2D) are needed [19]. The most beneficial effects in people with prediabetes or T2D would be an increase in MVPA concomitant with a decrease in SB. Interrupting extended periods of SB and replacing them with LIPA can benefit glucose control [20] and blood pressure [21]. To our knowledge, only few studies have evaluated the effect of randomised controlled trials (RCTs) with an outcome based on relative time and where all movement behaviours are considered [22–24].

The Sophia Step Study was a 2-year, three-armed pedometer-based intervention developed for primary care to support individuals with prediabetes or T2D to become physically active by regularly increasing their daily number of steps [25]. The 2-year effect of the intervention was assessed earlier using absolute time in each movement behaviour as the outcome [26]. However, because these analyses were conducted with absolute time in each movement behaviour and did not consider the relative time of the three behaviours, the results do not show the entire picture of how the behaviours change over time in relation to each other. Therefore, this study examines the effects of a 2-year pedometer-based intervention in people with prediabetes or T2D on relative time in different movement behaviours.

## Methods

### Study design and population

This study is a secondary analysis of the RCT Sophia Step Study [25]. Data were collected between 2013 and 2020. Participants were recruited from one rural and two urban primary care centres in Sweden by their diabetes nurse and randomised to one of the two intervention groups or the control group by sealed envelopes. All participants signed written informed consent prior to participation. Demographics and data on health conditions and medications were collected by a questionnaire and from patient medical records at baseline. The inclusion criteria were HbA1c > 39 mmol/mol or fasting glucose > 5.6 mmol/l, 40–80 years of age and fluency in Swedish. Exclusion criteria were myocardial infarction in the past 6 months, serum creatinine > 140 mmol/l, diabetic foot ulcer or risk of ulcer (severe peripheral neuropathy), patients newly prescribed insulin (< 6 months), other disease prohibiting physical activity, suffering repeated hypoglycaemia or severe hypoglycaemia in the past 12 months, very physically active according to the Stanford Brief Activity Survey [27] and those with no access to the internet.

### Intervention

The 2-year intervention was developed for the primary care context to support individuals with prediabetes or T2D to become physically active regularly. The RCT was three-armed with a multi-component intervention group that self-monitored their daily steps with a pedometer and registered them on a web-based platform. They were also offered group and individual counselling. The counselling was most intense for the first year (eight individual and ten group sessions) compared to the second year (two individual and two group sessions). A second group was offered a single-component intervention, including only self-monitoring and registration of daily steps. The third group was a control group receiving usual care. Details of the intervention and data collection can be found elsewhere [25].

### Measurement of movement behaviours

Time spent in MVPA, LIPA and SB was measured objectively using the ActiGraph GT1M accelerometer (ActiGraph, Pensacola, FL). Participants wore the accelerometer during waking hours. The accelerometer was placed on the participants’ lower back [28] for seven consecutive days at five time points (0 [baseline], 6, 12, 18 and 24 months). Additionally, the participants logged their daily wear time in a diary. The diaries were used to verify wear time and the number of valid days. The accelerations were sampled at 10 Hz and summed over 60 s using the software ActiLife v.6.13.4. Non-wear time was set to > 90 min (min) of consecutive zero counts, allowing for 2 min of nonzero counts [29]. Data were included for participants with ≥ 3 days and ≥ 10 h per day of valid wear time [30]. Wear time was allocated into activity categories based on count-based thresholds: SB < 100 counts per min (cpm) [31], LIPA 100–1951 cpm and MVPA ≥ 1952 cpm [32].

### Statistical analysis

All analyses were conducted using the R statistical system version 1.2.5019 and IBM SPSS version 27.0. Difference between groups at baseline were assessed with Chi-square test for categorical variables and ANOVA for continuous variables. Linear mixed models were used to investigate change in relative time in each movement behaviour (MVPA, LIPA, SB) in the three groups. Compositional means of time spent in MVPA, LIPA and SB were calculated by creating the geometric mean and summarising the behaviours to 100%. The daily time for each participant was expressed as a set of two isometric log-ratio (ilr) coordinates, including all relative information about the three compositional parts, as exemplified below for MVPA.

$$\mathrm{ilr}1 =\sqrt{\frac{2}{3}} \mathrm{ln}\frac{\mathrm{MVPA}}{\sqrt[2]{\mathrm{LIPA x SB}}}$$

$$\mathrm{ilr}2 =\sqrt{\frac{1}{2}} \mathrm{ln}\frac{\mathrm{SB}}{\sqrt[1]{\mathrm{LIPA}}}$$

A separate linear mixed model for relative time in each movement behaviour (MVPA, LIPA, SB) was conducted with the ilr_1_ variable for each behaviour as the outcome and 95% confidence intervals (CIs). This approach has been used elsewhere [22]. Participants were included as a random effect, age, randomisation group, time (as a categorical variable) and time by randomisation group interaction as fixed factors. Point estimates from the marginal means from the linear mixed models were back transformed into a percentage. The percentages from the three movement behaviours were adjusted to sum up to 100%. Contrast between baseline and 24 months were performed for relative time in each movement behaviour (MVPA, LIPA, SB).

## Results

**Fig. 1:**
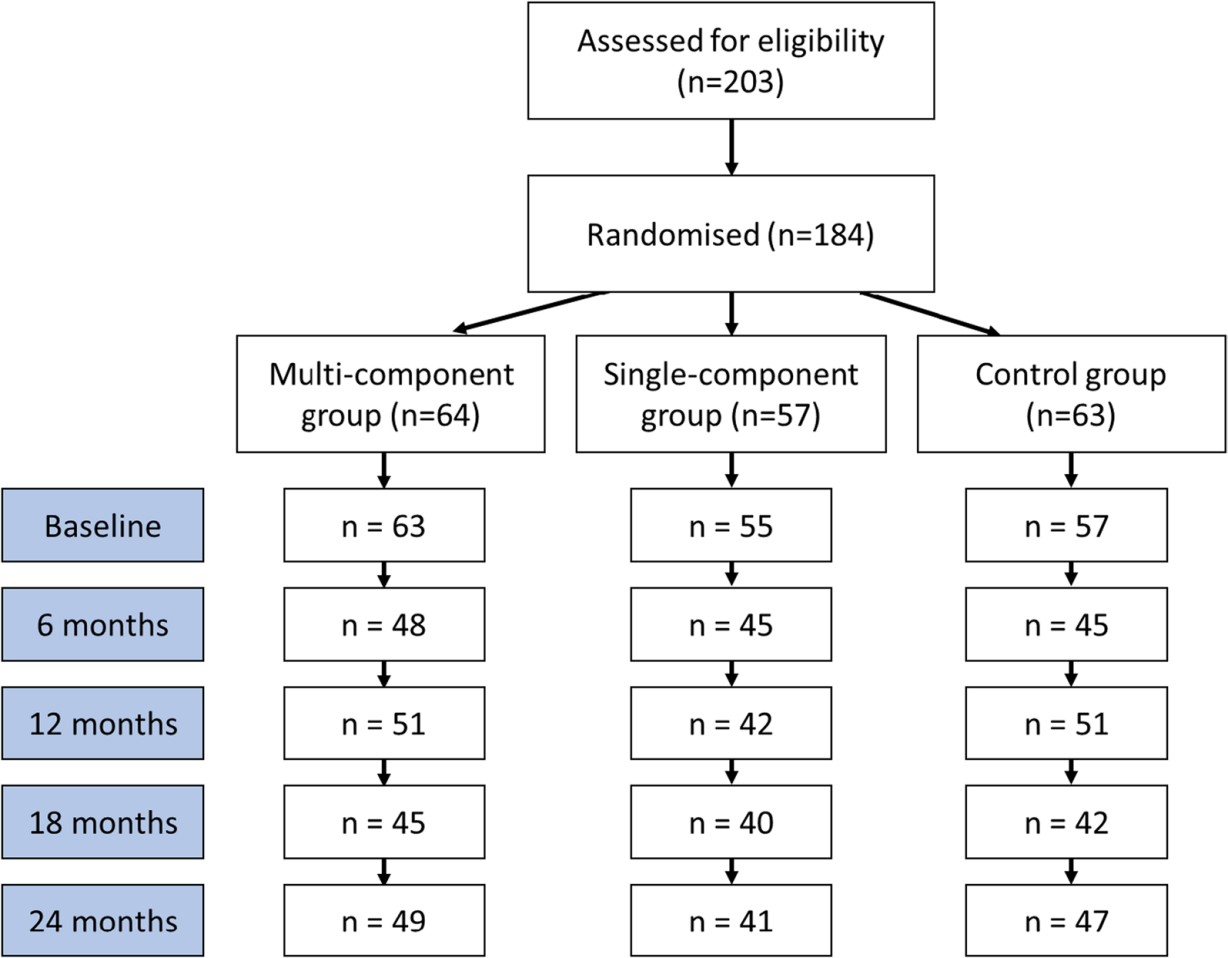
Flowchart of the number of participants with valid accelerometer data at each time point

In total 184 participants fulfilled the inclusion criteria and were randomised into the two intervention and control group. Figure 1 displays the number of participants with valid accelerometer data in each group at each time point. Table 1 presents the baseline participant characteristics by intervention group. Overall, 22% of the participants had prediabetes: mean (± SD) age was 64 ± 7.5 years, 41% were female and 47% had a university education. No statistically significant differences between the groups were found at baseline.

**Table 1:** Descriptive characteristics of the sample at baseline

	Total (n = 184)	Multi-component group (n = 64)	Single-component group (n = 57)	Control group (n = 63)	P-value*
Prediabetes, n (%)	40 (21.7)	13 (20.3)	10 (17.5)	17 (27.0)	0.431
Age (years), mean (SD)	64.3 (7.6)	64.2 (6.9)	65.5 (7.1)	63.3 (8.8)	0.287
Female, n (%)	76 (41.3)	28 (43.8)	24 (42.1)	24 (38.1)	0.802
University education, n (%)	86 (46.7)	28 (43.8)	25 (43.9)	33 (52.4)	0.390
Accelerometer wear time (min/day), mean (SD)	838 (74)	839 (92)	836 (60)	839 (65)	0.964
MVPA (min/day), mean (SD)	29.3 (23.7)	28.9 (20.5)	29.6 (25.0)	29.6 (25.9)	0.980
LIPA (min/day), mean (SD)	220.0 (65.4)	213.3 (59.7)	222.5 (72.3)	225.2 (64.9)	0.578
SB (min/day), mean (SD)	588.5 (84.9)	596.6 (89.6)	583.5 (91.1)	584.3 (73.3)	0.638
Relative time in MVPA (Ilr_1_), mean (SD)	− 2.39 (0.89)	− 2.36 (0.87)	− 2.39 (0.91)	− 2.41 (0.89)	0.947
Relative time in LIPA (Ilr_1_), mean (SD)	0.57 (0.44)	0.53 (0.46)	0.58 (0.44)	0.60 (0.60)	0.652
Relative time in SB (Ilr_1_), mean (SD)	1.82 (0.58)	1.83 (0.59)	1.81 (0.63)	1.81 (0.53)	0.983

*MVPA* moderate-to-vigorous intensity physical activity, *LIPA* light-intensity physical activity, *SB* sedentary behaviour, *Ilr*_*1*_ isometric log-ratio coordinate number 1, *SD* standard deviation. *P-value for difference between groups, assessed with Chi-square test for categorical variables and ANOVA for continuous variables

**Fig. 2:**
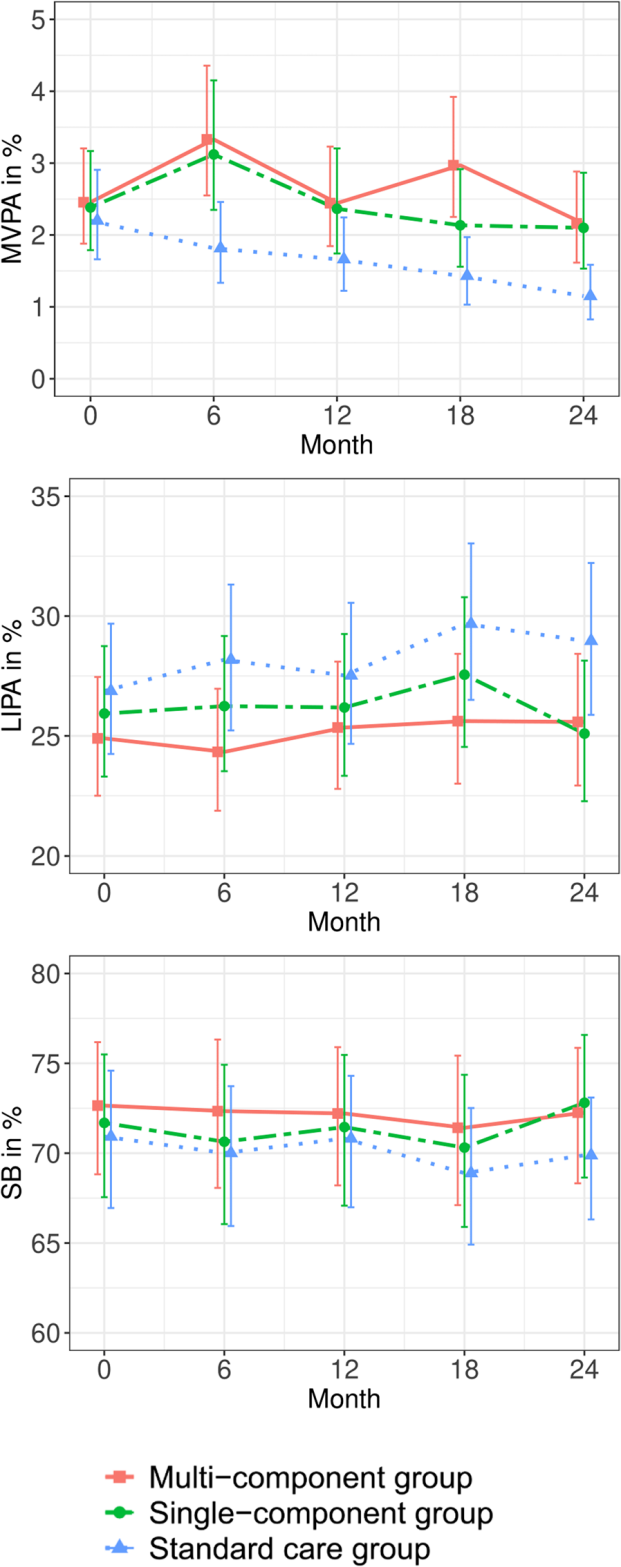
Changes in the relative time in per cent with 95% confidence intervals in movement behaviours over the 2-year study period. Values and related confidence intervals are based on the predicted group means from the linear mixed model analysis. *MVPA* moderate-to-vigorous intensity physical activity, *LIPA* light-intensity physical activity, *SB* sedentary behaviour

Table 2 shows the intervention effect over time between the two intervention groups and the control group. Overall, the effect over time favoured the intervention groups, although there was some variation in the magnitude of the effects at the different time points. In the multi-component group, compared to the control group, the intervention reached a statistically significant effect (interaction between time and group) on the relative time at 6 and 18 months in MVPA, LIPA and SB and for relative time at 24 months in MVPA and SB. In the single-component group compared with the control group, the intervention reached a statistically significant effect on relative time at 6 months in MVPA and SB and 24 months in MVPA and LIPA. For the control group, the within-group mean difference between baseline and 24 months showed a significant decrease in relative time in MVPA and an increase in relative time in LIPA and SB, see details in Table 3. No significant within-group changes were found between baseline and 24 months in the multi- or single-component groups. Table 4 lists relative time in per cent with 95% CIs for all movement behaviours for each group and measurement point based on the back-transformed point estimates from the linear mixed model. Figure 2 depicts changes in relative time in the movement behaviours over the 2-year study period (based on the numbers listed in Table 4).

**Table 2:** Intervention effects (interaction between time and group) from the linear mixed models for relative time in each behaviour (based on the isometric log-ratio) over time between the respective intervention groups and the control group

	Effect at 6 months (95% CI)	Effect at 12 months (95% CI)	Effect at 18 months (95% CI)	Effect at 24 months (95% CI)
Multi-component intervention vs control group				
Relative time in MVPA (Ilr_1_)	0.35 (0.07 to 0.63)	0.19 (− 0.08 to 0.47)	0.44 (0.15 to 0.72)	0.37 (0.09 to 0.65)
Relative time in LIPA (Ilr_1_)	− 0.16 (− 0.32 to − 0.01)	− 0.06 (− 0.20 to 0.09)	− 0.19 (− 0.35 to − 0.04)	− 0.15 (− 0.30 to 0.00)
Relative time in SB (Ilr_1_)	− 0.19 (− 0.35 to − 0.03)	− 0.14 (− 0.30 to 0.02)	− 0.24 (− 0.41 to − 0.08)	− 0.22 (− 0.38 to − 0.06)
Single-component intervention vs control group				
Relative time in MVPA (Ilr_1_)	0.32 (0.04 to 0.61)	0.19 (− 0.10 to 0.48)	0.22 (− 0.08 to 0.52)	0.37 (0.07 to 0.66)
Relative time in LIPA (Ilr_1_)	− 0.12 (− 0.28 to 0.03)	− 0.06 (− 0.22 to 0.09)	− 0.11 (− 0.27 to 0.05)	− 0.21 (− 0.37 to − 0.06)
Relative time in SB (Ilr_1_)	− 0.20 (− 0.37 to − 0.04)	− 0.13 (− 0.30 to 0.03)	− 0.12 (− 0.30 to 0.05)	− 0.16 (− 0.33 to 0.01)

*MVPA* moderate-to-vigorous intensity physical activity, *LIPA* light-intensity physical activity, *SB* sedentary behaviour. Analyses were conducted with one separate model for each outcome (relative time in each behaviour). CI = Confidence intervals. Ilr_1_ = Isometric log-ratio coordinate number 1

**Table 3:** Within group mean difference between baseline and 24 months

	Baseline to 24 months (95% CI)
Multi-component group	
Relative time in MVPA (Ilr_1_)	− 0.09 (− 0.27 to 0.09)
Relative time in LIPA (Ilr_1_)	0.05 (− 0.05 to 0.15)
Relative time in SB (Ilr_1_)	0.04 (− 0.07 to 0.15)
Single-component group	
Relative time in MVPA (Ilr_1_)	− 0.09 (− 0.32 to 0.14)
Relative time in LIPA (Ilr_1_)	− 0.02 (− 0.14 to 0.11)
Relative time in SB (Ilr_1_)	0.10 (− 0.02 to 0.23)
Control group	
Relative time in MVPA (Ilr_1_)	− 0.46 (− 0.67 to − 0.25)
Relative time in LIPA (Ilr_1_)	0.20 (0.09 to 0.31)
Relative time in SB (Ilr_1_)	0.26 (0.14 to 0.38)

*MVPA* moderate-to-vigorous intensity physical activity, *LIPA* light-intensity physical activity, *SB* sedentary behaviour, *Ilr*_*1*_ isometric log-ratio coordinate number 1, *CI* confidence intervals

**Table 4:** The relative time in percent with 95% confidence intervals for all physical activity behaviours for each group and measurement point

	Multi component group (n = 64)	Single component group (n = 57)	Control group (n = 63)
MVPA in % (95% CI)			
Month 0	2.5 (1.9 to 3.2)	2.4 (1.8﻿ to 3.2)	2.2 (1.7﻿ to 2.9)
Month 6	3.3 (2.6﻿ to 4.4)	3.1 (2.4﻿ to 4.2)	1.8 (1.3﻿ to 2.5)
Month 12	2.4 (1.8﻿ to 3.2)	2.4 (1.7﻿ to 3.2)	1.7 (1.2﻿ to 2.2)
Month 18	3.0 (2.3﻿ to 3.9)	2.1 (1.6﻿ to 2.9)	1.4 (1.0﻿ to 2.0)
Month 24	2.2 (1.6﻿ to 2.9)	2.1 (1.5﻿ to 2.9)	1.2 (0.8﻿ to 1.6)
LIPA in % (95% CI)			
Month 0	24.9 (22.5﻿ to 27.5)	25.9 (23.3﻿ to 28.7)	26.9 (24.2﻿ to 29.7)
Month 6	24.3 (21.9﻿ to 27.0)	26.2 (23.5﻿ to 29.2)	28.2 (25.2﻿ to 31.3)
Month 12	25.3 (22.8﻿ to 28.1)	26.2 (23.3﻿ to 29.3)	27.5 (24.7﻿ to 30.6)
Month 18	25.6 (23.0﻿ to 28.4)	27.6 (24.5﻿ to 30.8)	29.7 (26.5﻿ to 33.0)
Month 24	25.6 (22.9﻿ to 28.4)	25.1 (22.3﻿ to 28.1)	29.0 (25.9﻿ to 32.2)
SB in % (95% CI)			
Month 0	72.6 (68.8﻿ to 76.2)	71.7 (67.6﻿ to 75.5)	70.9 (67.0﻿ to 74.6)
Month 6	72.3 (68.1﻿ to 76.3)	70.6 (66.1﻿ to 74.9)	70.0 (65.9﻿ to 73.7)
Month 12	72.2 (68.2﻿ to 75.9)	71.5 (67.1﻿ to 75.5)	70.8 (67.0﻿ to 74.3)
Month 18	71.4 (67.1﻿ to 75.4)	70.3 (65.9﻿ to 74.4)	68.9 (64.9﻿ to 72.5)
Month 24	72.3 (68.3﻿ to 75.9)	72.8 (68.6﻿ to 76.6)	69.9 (66.3﻿ to 73.1)

*MVPA* moderate-to-vigorous intensity physical activity, *LIPA* light-intensity physical activity, *SB* sedentary behaviour, *CI* Confidence intervals

## Discussion

This study investigated the effects of a 2-year pedometer-based intervention in people with prediabetes or T2D on relative time in movement behaviours. We found an intervention effect on all behaviours targeted in both intervention groups, although some variations in the magnitude and time point of the effects were noted. In the multi-component group an effect for all targeted behaviours was seen at 6 and 18 months; for the MVPA and SB behaviours, an effect was found at 24 months. In the single-component group an effect occurred at 6 months for MVPA and SB and 24 months for MVPA and LIPA. At these time points, the estimated marginal means (transformed into per cent) ranged from 0.9 to 1.5% of more MVPA, 1.9 to 3.9% of less LIPA and from 0.5% of less SB to 1.7 more SB in the intervention groups compared to the control group. Assuming that the participants were awake 16 h per day, these differences in percentages correspond to 9 to 15 min/day in more MVPA, 18 to 37 min/day in less LIPA and 5 min/day less SB to 16 min/day more SB in the intervention groups compared to the control group.

The magnitude of our results seems to be larger and longer lasting compared to other pedometer-based interventions evaluating physical activity behaviours using absolute time in people with prediabetes or T2D [33–35]. These studies have shown mixed results ranging from no effect to a small effect (MVPA + 3.5 min/day, LIPA + 5.1 to + 14.1 min/day, SB − 5.2 to − 14.4 min/day) at 6 or 12 months. Yet, the effects were not sustained in the long run (up to 48 months). Moreover, systematic reviews have concluded that pedometer-based interventions, often together with counselling, can positively impact increasing physical activity in people with T2D [10, 13]. However, the effects were only evident during the intervention period [13].

Moreover, the within-group mean difference between baseline and 24 months showed that the control group decreased the relative time in MVPA, which was compensated by increased relative time in LIPA and SB. This trend was not observed in the multi-component or single-component groups. Another pedometer-based intervention found an increase in SB and LIPA. At the same time, MVPA decreased in the control group [34], suggesting that pedometer-based interventions can help prevent unhealthy physical activity behaviours from developing over time.

The present findings should be viewed in relation to our previous study, in which our outcome measures were absolute time of each behaviour. This earlier study found significant intervention effects only in MVPA: at all time points in the multi-component group and at the 6-month time point in the single-component group. No intervention effects were found in LIPA or SB [26]. For people with prediabetes or T2D, increased time in MVPA and decreased time in SB seem necessary to improve glucose control [6, 36], raising the possibility that using relative time could be a preferred option to present a more thorough conceptualisation of how all movement behaviours are affected by interventions.

Given all movement behaviours taken together, the most beneficial change can be seen in the multi-component group at 6 and 18 months, with increased relative time in MVPA and decreased relative time in LIPA and SB. However, the improvements seem to return to baseline values at 24 months. The individual and group counselling interventions were most intense during the first year, indicating the need for continuous follow-up [37].

Results have varied in other population groups depending on whether relative or absolute time was used. Chastin et al., for instance, found a difference of about 10 min in MVPA when they compared relative to absolute mean times in cross-sectional data [17]. Gupta et al. analysed time spent in SB, standing and physical activity during work and leisure in cross-sectional data using relative and absolute time approaches. They concluded that effect sizes could differ, favouring the relative time approach, even if the results with both techniques were significant [38]. Other RCTs have used both relative and absolute time or absolute time alone to evaluate the effect of physical activity interventions. Pasanen et al. did not find any effect of an activity tracker intervention when they used relative time at 6 months [23] or absolute time at 12 months [39, 40]. Another RCT that reduced office workers’ sitting time found a significant effect when a relative and absolute time approach was used [24, 41]. A recent RCT examined the effect of physical activity and SB interventions on office workers. The authors could not find any effect on relative time in physical activity behaviours [22].

### Strengths and limitations

The main strengths of this study are the longitudinal design, the use of device-based measures for movement and having relative time in all three behaviours as outcomes, as this considers the complex nature of physical activity [17]. A limitation of this study was that we did not measure sleep time, making it impossible to address 24-h movement behaviour patterns. However, this would not affect the intervention effect between the groups due to equal groups by the RCT design of the study. Also, the baseline values indicate that the participants were already active at baseline (mean MVPA in the entire group was 29.3 min per day). Thus, we might have failed to reach the inactive people who could benefit most from this type of intervention. Using accelerometers includes limitations, such as the failure to detect activities (e.g., bicycling and muscle-strengthening activities). Finally, the fact that participants were aware of being measured can affect their behaviour during the measurement period, leading to a failure to capture actual behaviours.

## Conclusion

In this study relative time was used to evaluate the effects of a physical activity intervention. The findings show a beneficial effect on all movement behaviours over time in both intervention groups, with a more pronounced effect in the multi-component group. The control group had a negative trend in change and compensation among the behaviours over time, implying that counselling should be a key component in pedometer-based interventions. Using relative time as the outcome measure provides a more comprehensive assessment of the pattern of change in physical activity interventions, than using absolute time.

## Data Availability

The datasets generated or analysed during the current study are not publicly available because the data can be traced back to the study participants. According to Swedish and EU data legislation, access can only be granted upon a reasonable request. The request should be addressed to the PI and will be handled on a case‑by‑case basis. Data sharing will be regulated via a data transfer and use agreement with the recipient.
